# A Novel Repetition Frequency-Based DNA Encoding Scheme to Predict Human and Mouse DNA Enhancers with Deep Learning

**DOI:** 10.3390/biomimetics8020218

**Published:** 2023-05-23

**Authors:** Talha Burak Alakuş

**Affiliations:** Department of Software Engineering, Faculty of Engineering, Kırklareli University, 39100 Kırklareli, Turkey; talhaburakalakus@klu.edu.tr

**Keywords:** DNA enhancer, DNA encoding scheme, deep learning, artificial intelligence

## Abstract

Recent studies have shown that DNA enhancers have an important role in the regulation of gene expression. They are responsible for different important biological elements and processes such as development, homeostasis, and embryogenesis. However, experimental prediction of these DNA enhancers is time-consuming and costly as it requires laboratory work. Therefore, researchers started to look for alternative ways and started to apply computation-based deep learning algorithms to this field. Yet, the inconsistency and unsuccessful prediction performance of computational-based approaches among various cell lines led to the investigation of these approaches as well. Therefore, in this study, a novel DNA encoding scheme was proposed, and solutions were sought to the problems mentioned and DNA enhancers were predicted with BiLSTM. The study consisted of four different stages for two scenarios. In the first stage, DNA enhancer data were obtained. In the second stage, DNA sequences were converted to numerical representations by both the proposed encoding scheme and various DNA encoding schemes including EIIP, integer number, and atomic number. In the third stage, the BiLSTM model was designed, and the data were classified. In the final stage, the performance of DNA encoding schemes was determined by accuracy, precision, recall, F1-score, CSI, MCC, G-mean, Kappa coefficient, and AUC scores. In the first scenario, it was determined whether the DNA enhancers belonged to humans or mice. As a result of the prediction process, the highest performance was achieved with the proposed DNA encoding scheme, and an accuracy of 92.16% and an AUC score of 0.85 were calculated, respectively. The closest accuracy score to the proposed scheme was obtained with the EIIP DNA encoding scheme and the result was observed as 89.14%. The AUC score of this scheme was measured as 0.87. Among the remaining DNA encoding schemes, the atomic number showed an accuracy score of 86.61%, while this rate decreased to 76.96% with the integer scheme. The AUC values of these schemes were 0.84 and 0.82, respectively. In the second scenario, it was determined whether there was a DNA enhancer and, if so, it was decided to which species this enhancer belonged. In this scenario, the highest accuracy score was obtained with the proposed DNA encoding scheme and the result was 84.59%. Moreover, the AUC score of the proposed scheme was determined as 0.92. EIIP and integer DNA encoding schemes showed accuracy scores of 77.80% and 73.68%, respectively, while their AUC scores were close to 0.90. The most ineffective prediction was performed with the atomic number and the accuracy score of this scheme was calculated as 68.27%. Finally, the AUC score of this scheme was 0.81. At the end of the study, it was observed that the proposed DNA encoding scheme was successful and effective in predicting DNA enhancers.

## 1. Introduction

The region just opposite the point where a gene begins to be read is called the promoter [1]. A promoter is a short region of DNA that increases the rate of transcription of genes in a gene cluster [2]. The promoter region contains DNA sequences that control the expression of genes. In eukaryotes, apart from promoter regions, DNA regions called enhancers also affect gene expression. Although these enhancers are far from the transcription start point, they are close to each other in three-dimensional space [3]. Enhancers, promoters, silencers, and insulators are expressed as cis-regulatory and play important roles in the gene expression process.

Predicting enhancers plays an important role in the discovery of biological activities in organisms. Today, with the development and advancement of technology, there are various experimental approaches used to predict DNA enhancers. Examples of these approaches are DNaseI-seq (DNAseI-digested chromatin sequencing), ChIP-Seq (chromatin immunoprecipitation sequencing), RNA-Seq (RNA sequencing), FAIRE-Seq (formaldehyde-assisted isolation of regulatory elements sequencing) [4,5,6]. Identifying the enhancers found in the gene is a difficult and tedious task. The main reason for this is the partially complex coding structures of the enhancers, their presence in other genes, and the absence of a unique code [7,8]. In addition, these approaches require a laboratory, which causes them to be ineffective in terms of both time and cost.

To prevent these problems, the importance of computation-based approaches has increased recently, and deep-learning-supported systems have been used in this field. To use deep learning methods, DNA sequences must be converted to numerical representations. In this case, the performance of computation-based approaches depends on the digital encoding scheme used and the deep learning model applied [9]. This dependence on computation-based approaches leads to poor and inconsistent prediction performance [10]. Therefore, it is necessary to develop novel encoding schemes and use consistent approaches.

In this study, a novel DNA encoding scheme is proposed to avoid the mentioned problems and is used to predict DNA enhancers. The study consisted of two different scenarios and a total of four stages. In the first stage, DNA enhancer data of human and mouse genomes were obtained. The VISTA Enhancer Browser dataset was used for this process. In the second stage, DNA data were converted to numerical representations with various DNA encoding schemes and the proposed repetition-frequency-based DNA encoding scheme. In this stage, EIIP (electron–ion interaction potential), integer number, and atomic number DNA encoding schemes were used. In the third stage, a deep learning model was designed and BiLSTM (bidirectional long short-term memory) model was used. In the last stage, the performances of DNA encoding schemes were determined, and accuracy, precision, recall, F1-score, CSI (classification success index), G-mean (geometric mean), MCC (Matthew’s correlation coefficient), Kappa coefficient, and AUC (area under curve) scores were used for this. These stages were performed for both scenarios. In the first scenario, it was predicted whether the DNA enhancers were human or mouse. In the second scenario, it is predicted whether the enhancers belong to humans or mice, and in addition, whether they are enhancers. The highlights of the study can be summarized as follows:

- In this study, a novel DNA encoding scheme was proposed and this scheme was used for the prediction of DNA enhancers.
- In this study, DNA enhancers were analyzed and predicted by various DNA encoding schemes. For the first time in this study, EIIP, integer number, and atomic number DNA encoding schemes were applied to this field.
- For the first time in this study, the BiLSTM deep learning model was applied to the mentioned DNA encoding schemes.

The organization of the study is as follows: In the second section, a few studies in this field were examined. The methods used in those studies and the performances of the classifiers were given. In the third section, the data set and DNA encoding schemes were mentioned. In addition to these, the proposed DNA encoding scheme was explained in detail in this section. Finally, the deep learning model and evaluation metrics were specified. In the fourth section, the results of the application were given, and the discussion was carried out. Moreover, the advantages and disadvantages of the study were also mentioned. In the last section, the study was summarized and the contributions of the study to the literature and future study were mentioned.

## 2. Related Works

In this section, various prediction studies with DNA enhancers and deep learning models were mentioned. When the literature is examined, although the scarcity of studies in this field is revealed, a new field of study makes these studies valuable. In the study [10], researchers predicted DNA enhancers using the DBN (deep belief network) deep learning model. In the study, the VISTA Enhancer Browser dataset was used, and the prediction process was performed on 741 human DNA enhancers. DNA sequences were converted to the numerical representations by the k-mer DNA encoding scheme, and the k value was determined as two, three, and four in the study. A total of 168 k-mer features were used in the study. Then, classification was performed with the DBN network, and the performance of the classifier was measured only with the accuracy evaluation criterion. At the end of the study, an accuracy score of 92.0% was obtained. In study [11], the researchers created a hybrid deep learning model and predicted DNA enhancers. In the study, DNA sequences were converted to numbers by a binary hot-coding scheme and prepared for classification. The data used in the study were obtained from the VISTA Enhancer Browser dataset and a total of 1747 experimentally proven human genome intron elements were used. For the classification process, a hybrid deep learning model was designed and for this, CNN (convolutional neural network) and DLSTM (deep long short-term memory) architectures were combined. In addition to these, KNN (K nearest neighbor), SVM (support vector machines), and RF (random forest) machine learning methods were also used in the study. The performance of the classifiers was determined by the precision, accuracy, specificity, and MCC evaluation metrics. At the end of the study, the highest accuracy score was determined with the hybrid deep learning model and the result was 89.7%. In another study, researchers predicted DNA enhancers from chromatin strands using the RF algorithm [12]. H1 and IMR90 datasets were used in the study. The parameters of the classifier were determined by using ROC (receiver characteristic curve) and the most suitable parameters were selected. In this way, the optimum number of trees was decided. The performance of the classifier was determined by the misclassification rate, and at the end of the study, a misclassification rate of about 0.05 was achieved. In study [13], the researchers predicted DNA enhancers using the word embedding method and deep learning. DNA sequences were encoded by the GAN (generative adversarial networks) method and evaluated for use in the classifier. The deep learning models used in the study were based on the BiLSTM and CNN-LSTM methods. The performance of the classifiers was measured with accuracy, precision, recall, and MCC evaluation criteria. At the end of the study, an accuracy score of 93.95% was observed. In study [14], researchers developed a deep learning algorithm-based model and predicted DNA enhancers. In the study conducted on H1 cells, a total of nine features were obtained from DNA sequences, including histone modifications, TFs and cofactors, chromatin accessibility, transcription, DNA methylation, CpG islands, evolutionary conversion, sequence signatures, and TF binding sites. For the classification process, DNN (deep neural networks) and HMM (hidden Markov model) were used and the performance of the classifiers was determined by accuracy, recall, precision, and F1-score values. At the end of the study, an accuracy score of 96.82% was obtained with the proposed deep learning model. When the literature review was examined, it was observed that DNA enhancers were successfully predicted by deep learning algorithms. Based on these achievements, BiLSTM, one of the deep learning types, was used in this study and its results were analyzed based on DNA encoding schemes.

## 3. Materials and Methods

In this section, the dataset and DNA encoding schemes used in the study were mentioned. In addition to these, the proposed novel repetition frequency-based scheme was also explained. Finally, information about BiLSTM and the evaluation metrics were given.

### 3.1. Data Set

In this study, the Vista Enhancer Browser dataset was used [15]. A key resource for experimentally verified human and mouse non-coding regions with gene enhancer activity as determined in transgenic mice is the VISTA Enhancer Browser. The majority of these non-coding elements were chosen for testing because of their high conservation in other vertebrates or because of epigenomic proof (ChIP-Seq) of potential enhancer marks. This freely accessible website offers the outcomes of an in vivo enhancer screen. A total of 3321 in vivo tested elements have been recorded as of 7 April 2023, including 1699 elements having enhancer activity. Within the scope of the study, both enhancer-containing and non-enhancer DNA sequences of both humans and mice were obtained from this dataset. In the study, these sequences were separated, and the type of enhancers was determined. In addition to these, a distinction was made whether the sequence was an enhancer or not.

### 3.2. DNA Encoding Schemes

In this study, EIIP, integer number, and atomic number DNA encoding schemes were used. Although the integer number encoding scheme is evaluated in the fixed category, EIIP and atomic number encoding schemes are included in the biochemical-based encoding schemes.

#### 3.2.1. Integer Number DNA Encoding Scheme

The integer number DNA encoding scheme is a widely used method [16,17,18]. In this scheme, arbitrary values of 1, 3, 2, and 4 are assigned to bases A, C, G, and T, which are nucleotides in DNA sequences, respectively [19]. DNA sequences are encoded according to the expression given in Equation (1).

(1) $$\mathrm{Integer}\;\mathrm{Representation}=x↔n,\;x\;\epsilon \;\left\{A,\;C,\;G,\;T\right\},\;n\;\epsilon \;\left\{1,\;3,\;2,\;4\right\}$$

In Equation (1), *n* represents integers, whereas *x* represents bases. According to the values in Equation (1), a DNA sequence as S(*n*) = [ACACCCAGGT] is encoded as C(*n*) = [1 3 1 3 3 3 1 2 2 4] by integer number DNA encoding scheme.

#### 3.2.2. Atomic Number DNA Encoding Scheme

In the atomic number DNA encoding scheme, DNA bases are expressed according to their atomic number. In this scheme, bases are converted into an atomic indicator and bases are valued according to these indicators. This scheme was first proposed and used to determine the fractal size difference between humans and chimpanzees [20]. In this scheme, A takes 70, C gets 58, G takes 78 and T gets 66. According to the expression given in Equation (2), DNA sequences are converted to numerical representations.

(2) $$\mathrm{Atomic}\;\mathrm{Number}\;\mathrm{Representation}=x↔n,\;x\;\epsilon \;\left\{A,\;C,\;G,\;T\right\},\;n\;\epsilon \;\left\{70,\;58,\;78,\;66\right\}$$

In Equation (2), *n* denotes atomic numbers, while *x* denotes bases. According to the values in Equation (2), a DNA sequence as S(*n*) = [ACACCCAGGT] is encoded as C(*n*) = [70 58 70 58 58 58 70 78 78 66] by atomic number DNA encoding scheme.

#### 3.2.3. EIIP DNA Encoding Scheme

The EIIP encoding scheme has been proposed as an alternative to the Voss DNA encoding scheme and is a frequently used method in many FFT (fast Fourier transform) based GSP (genomic signal processing) applications [21,22]. In this scheme, the so-called potential energies of free electrons are assigned to the bases in the DNA sequence and encoded. In the EIIP DNA encoding scheme, the A base is 0.1260, the C base is 0.1340, the G base is 0.0806, and the T base is 0.1335. According to the expression given in Equation (3), DNA sequences are converted.

(3) $$\mathrm{EIIP}\;\mathrm{Representation}=x↔n,\;x\;\epsilon \;\left\{A,\;C,\;G,\;T\right\},\;n\;\epsilon \;\left\{0.1260,\;0.1340,\;0.0806,\;0.1335\right\}$$

In Equation (3), *n* specifies EIIP values, while *x* denotes bases. According to the values in Equation (3), a DNA sequence as S(*n*) = [ACACCCAGGT] is encoded as C(*n*) = [0.1260 0.1340 0.12600.1340 0.1340 0.1340 0.1260 0.0806 0.0806 0.1335] by EIIP DNA encoding scheme.

### 3.3. A Novel DNA Encoding Scheme Based on Base Repetition Frequency

TF (term frequency) is a method that is frequently used in NLP (natural language processing) studies and is used to convert words into numerical expressions [23,24]. TF expression in NLP is obtained by dividing a word by the total number of words. For instance, if there are 5 bananas in a 100-word document, the TF value of the term banana is 0.05 (5/100). In this way, in NLP studies, applications consider 0.05 instead of banana expression. In the TF approach, the threshold value is generated by the algorithm itself rather than the user’s choice [23]. This causes the method to change according to the given input value and to be dynamic. Considering the performances of the TF method in NLP studies, a similar approach was used in this study and the bases in the DNA sequence were converted into numerical expressions according to this approach. In the proposed DNA encoding scheme, the process is performed according to the length of the sequence and the frequency of the bases in that sequence. Since the proposed method is based on the base frequency in the DNA sequence, it is named BFDNA (base frequency DNA). In Equation (4), the encoding formula of the BFDNA DNA encoding scheme is given.

(4) $$\mathrm{BFDNA}=\frac{\mathrm{num}.\;\mathrm{of}\;\mathrm{times}\;X\;\mathrm{base}\;\mathrm{occurs}\;\mathrm{in}\;a\;\mathrm{DNA}\;\mathrm{sequence}}{\mathrm{Total}\;\mathrm{sequence}\;\mathrm{length}}$$

In Equation (4), the *X* value represents the bases found in the DNA sequence. DNA sequences consist of four different bases, A, C, G, and T in total. Here the *X* value is calculated for each base. According to the values in Equation (4), a DNA sequence S(*n*) = [ACACCCAGGT] is converted into numerical expressions step by step with the BFDNA encoding scheme as follows:

- For A base: ${\mathrm{BFDNA}}_{\left(A\right)}=\frac{3}{10}=0.3$
- For C base: ${\mathrm{BFDNA}}_{\left(C\right)}=\frac{4}{10}=0.4$
- For G base: ${\mathrm{BFDNA}}_{\left(G\right)}=\frac{2}{10}=0.2$
- For T base: ${\mathrm{BFDNA}}_{\left(T\right)}=\frac{1}{10}=0.1$

Since the length of the sequence is 10, the total number of each base is divided by 10. There were 3 A bases in total in the sequence; the BFDNA value of this base was 0.3, and since there were 4 C bases, the BFDNA value of this base was calculated as 0.4. Furthermore, there are 2 G bases and 1 T base in total from the sequence; the BFDNA values of these bases were obtained as 0.2 and 0.1, respectively. According to the calculation step, a DNA sequence S(*n*) = [ACACCCAGGT] is encoded with the BFDNA DNA encoding scheme as C(*n*) = [0.3 0.4 0.3 0.4 0.4 0.4 0.3 0.2 0.2 0.1]. One of the most important features of the proposed DNA encoding scheme is that it has a dynamic structure. Almost all the other DNA encoding schemes found in the literature and used in this study have a static structure. Regardless of the length of the sequence, the bases take the same value. However, there is no such structure in the proposed scheme. Although other DNA encoding schemes used in the study consist of fixed values, the BFDNA scheme does not include fixed values. A comparison of DNA encoding schemes used for DNA sequences of the same species is given in Table 1.

As seen in the example given in Table 1, the bases in the two DNA sequences take the same values in the integer number, atomic number, and EIIP encoding schemes. In the integer number DNA encoding scheme, the C base in the first DNA sequence was assigned as 3, whereas the C base in the second DNA sequence was expressed with the value 3. Similar inferences can be made with atomic numbers and EIIP DNA encoding schemes. However, in the proposed BFDNA encoding scheme, the value of the C base in the first DNA sequence was 0.4, whereas this value was calculated as 0.2 in the second DNA sequence. This shows that the proposed approach is not fixed and has a dynamic structure. As the sequence length increases, this difference between DNA encoding schemes becomes more pronounced. In addition, degeneration is observed over time in encoding schemes that consist of fixed values and cause the loss of information [25]. In order to prevent such information loss and to be used to predict DNA enhancers, the BFDNA encoding scheme, which has a dynamic structure, has been proposed and applied in the study.

### 3.4. BiLSTM Deep Learning Model

**Figure 1:**
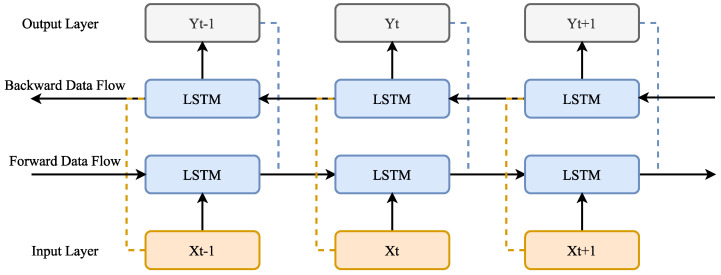
Structure of BiLSTM deep learning model.

Deep learning is a form of machine learning that is now employed successfully. One of the main factors contributing to its popularity is the ease and speed with which data can be gathered, as well as the ability to meet the hardware requirements for data analysis [26]. Moreover, dealing with complicated and huge data sets has a role in this regard. The method of feature extraction is deep learning’s main advantage over machine learning. Deep learning uses an adaptive strategy, whereas machine learning-based methods manually extract essential information [27]. Manual feature extraction is challenging and time-consuming due to the quantity of data or the complexity of the data collection [28]. Deep learning is now utilized in nearly every sector thanks to these benefits. Studies in the disciplines of biomedicine [29,30], bioinformatics [31], object identification [32], robotics [33], and energy [34] have all used deep learning. The success of deep learning in various fields has laid the groundwork for the use of deep learning in this study. For this reason, BiLSTM, one of the deep learning methods, was used in this study. The BiLSTM deep learning model is a kind of RNN (recurrent neural network). It is formed by adding another LSTM layer to the LSTM layer. Sequential structures such as time and text series make better use of LSTM [35]. Moreover, the biological sequence analysis challenge has been successfully solved using LSTM on a large scale because of its structure which takes advantage of historical data [36]. This model can remember information about the past as well as the future because of this structure. By using the BiLSTM architecture, both forward and backward calculations are performed concurrently, and the output is produced by merging the data that was discovered as a result of both sets of computations. Due to this, processing sequential data and time series benefits from the usage of information in two directions. Although one of the two LSTM units in the structure of BiLSTM processes the information backward, the other processes the input. One of the biggest reasons for using the BiLSTM method in the study is that each element of an input sequence contains data from the past and the present. For this reason, by integrating LSTM layers from both directions, BiLSTM can generate an output that is more meaningful [37]. Furthermore, each component (A, C, G, T) in the sequence (DNA sequence) will provide a different output from the BiLSTM. Therefore, the BiLSTM model is useful for some bioinformatics applications such as predicting protein–protein interactions, determining protein structures, and predicting DNA promoters [38]. Due to the performances demonstrated in bioinformatics studies and the advantages of its structure, BiLSTM was used in this study. A general schematic of the BiLSTM deep learning architecture is given in Figure 1.

### 3.5. Evaluation Criteria

In this study, the performances of deep learning models were determined by the evaluation criteria of accuracy, precision, recall, F1-score, AUC, and confusion matrix. The confusion matrix is a kind of table used to show the performance of a classification algorithm. Performance is based on the determination of training data by testing. There are four different parameters in the confusion matrix: FP stands for false positive, TN for true negative, TP for true positive, and TN for true negative. The TP expression indicates that the predicted value is positive and correct. In addition, the TN expression indicates that the estimated value is negative and correct. In addition, FP indicates that the value is positive but false, while FN indicates that the value is negative and false. Accuracy, F1-score, precision, and recall values are based on the calculation of these given expressions into various equations. The calculation of these operations is shown between Equations (5) and (8).

(5) $$\mathrm{Accuracy}=\frac{\mathrm{TP}+\mathrm{FN}}{\mathrm{TP}+\mathrm{TN}+\mathrm{FP}+\mathrm{FN}}$$

(6) $$\mathrm{Precision}=\frac{\mathrm{TP}}{\mathrm{TP}+\mathrm{FP}}$$

(7) $$\mathrm{Recall}=\frac{\mathrm{TP}}{\mathrm{TP}+\mathrm{FN}}$$

(8) $$F1-\mathrm{Score}=2\times \left(\frac{\mathrm{Precision}\times \mathrm{Recall}}{\mathrm{Precision}+\mathrm{Recall}}\right)$$

In addition, the AUC score is also an important evaluation criterion. The AUC score gives the area under the ROC (receiver characteristic curve). The AUC score usually indicates how good the model is at separating classes. In addition, it is also used to determine the best threshold [39]. The ranges given in Table 2 are taken into account when interpreting the AUC score.

AUC score of 0.49 and below indicates that the classification process was not performed. In addition, if the AUC score is between 0.50 and 0.69, it is considered a poor classification. AUC score between 0.70 and 0.79 indicates an acceptable classifier, whereas a value between 0.80 and 0.89 indicates excellent classification. Values of 0.90 and above indicate an extraordinary classification.

CSI is an uncommon evaluation criterion that uses the performance of the classification process. The positive class is the only thing the CSI concentrates on [41]. It is calculated by the formula given in Equation (9).

(9) CSI= Precision+True Positive Rate−1

The CSI value varies between −1 and 1. A value of −1 means that all predictions are false positive or false negative [41]. A value of 1 indicates that the predictions are excellent. A CSI value of 0 means that the results are random predictions.

G-mean is an evaluation criterion that measures the balance between the performance of classes. It is determined by the formula in Equation (10).

(10) $$\mathrm{Gmean}=\sqrt{\mathrm{Recall}\times \mathrm{Specificity}}$$

A high value indicates that the risk of over-fitting for negative classes is low, and the risk of under-fitting for positive classes is low.

MCC is an evaluation metric used to determine the performance of a classification model. It is an approach that is effective when the number of data between classes is unbalanced (uneven) [42]. It is calculated by the formula given in Equation (11).

(11) $$\mathrm{MCC}=\frac{\left(\mathrm{TP}\times \mathrm{TN}-\mathrm{FP}\times \mathrm{FN}\right)}{\sqrt{\left(\mathrm{TP}+\mathrm{FP}\right)\times \left(\mathrm{TP}+\mathrm{FN}\right)\times \left(\mathrm{TN}+\mathrm{FP}\right)\times \left(\mathrm{TN}+\mathrm{FN}\right)}}$$

The MCC value approaching 0 indicates that the classification model is unsuccessful, whereas the value approaching 1 indicates that this model is effective. It is an evaluation criterion mostly used in binary classification problems [43].

Cohen’s Kappa coefficient is an evaluation metric used to measure agreement between classes. It is calculated by the formula given in Equation (12).

(12) $$\mathrm{Kappa}\;\mathrm{coefficient}=\frac{2\times \left(\mathrm{TP}\times \mathrm{TN}-\mathrm{FP}\times \mathrm{FN}\right)}{\left(\mathrm{TP}+\mathrm{FP}\right)\times \left(\mathrm{FP}+\mathrm{TN}\right)+\left(\mathrm{TP}+\mathrm{FN}\right)\times \left(\mathrm{FN}+\mathrm{TN}\right)}$$

The Kappa coefficient varies from 0 to 1. Kappa values and interpretation of these values are given in Table 3.

**Figure 2:**
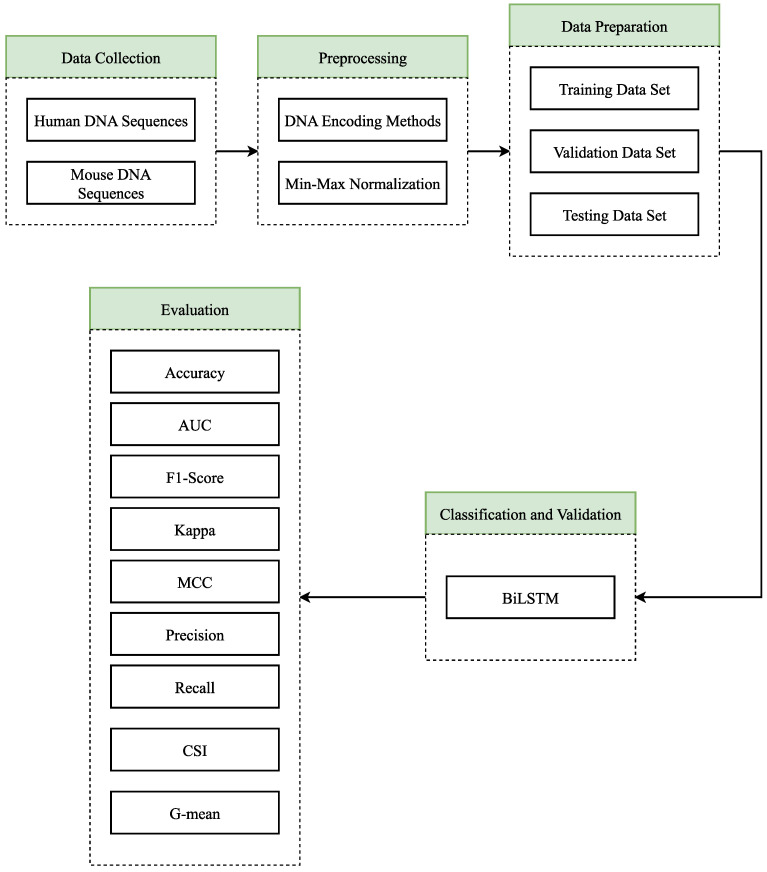
Flowchart of the study.

A kappa value of 0 indicates that there is no agreement between the classes, whereas values between 0.10 and 0.20 indicate that there is slight agreement. A sufficient agreement is observed if the kappa coefficient is between 0.21 and 0.40. A moderate agreement is observed when the value is between 0.41 and 0.60, whereas a substantial agreement is available when the value is between 0.61 and 0.80. The kappa value between 0.81 and 0.99 indicates that the model makes an almost perfect agreement. In addition, a kappa coefficient of 1 indicates that a perfect agreement is observed. The flow chart of the study is given in Figure 2.

According to the workflow given in Figure 2, human and mouse DNA sequences were obtained in the first step (Data Collection). Although some of these sequences consist of DNA enhancers, some do not have DNA enhancers. Then (Preprocessing) these sequences were converted into numerical expressions with both the proposed DNA encoding scheme and various DNA encoding schemes. After the sequences were converted to numerical expressions, min–max normalization was performed, and the sequences were normalized. In the third step (Data Preparation), the data were prepared, and the data set was divided into three as training, testing, and validation. In the fourth step (Classification and Validation), the BiLSTM deep learning model was designed, and the training process was carried out with the training data. Then, the parameters of the classifier were adjusted with the validation data set, and the most effective parameters were selected. In the last step (Evaluation), the performance of the classifier was tested on the test data set and the performance of each DNA encoding scheme was measured with accuracy, precision, recall, F1-score, CSI, G-mean, MCC, Kappa coefficient, and AUC scores.

## 4. Application Results

In this study, the application was carried out through two different scenarios. In the first scenario, it was predicted whether the DNA enhancers belonged to humans or mice, whereas in the second application, it was predicted whether there was a DNA enhancer and, if any, whether it belonged to a human or mouse. At the end of the prediction process, the accuracy, precision, recall, F1-score, CSI, G-mean, MCC, Kappa coefficient, and AUC scores of each DNA encoding scheme were calculated.

### 4.1. Predicting Human and Mouse Enhancers

In the study, a novel DNA encoding scheme was proposed to predict human and mouse DNA enhancers and compared with various DNA encoding schemes. In this direction, the performances of DNA encoding schemes were determined by using the BiLSTM deep learning model, with accuracy, precision, recall, F1-score, CSI, G-mean, MCC, Kappa coefficient, and AUC scores. The hyperparameters of the developed BiLSTM model are as follows:

- DNA sequences used for training data in the input layer were evaluated.
- In the second layer, 256-unit BiLSTM was used, and the activation function was determined as SeLU (scaled exponential linear unit).
- Dropout was done and 15% of the data was discarded.
- Then the 128-unit BiLSTM was used and the activation function SeLU was chosen.
- Again, dropout was selected and 20% of the data was forgotten.
- Then the 64-unit BiLSTM was used and the activation function SeLU was chosen.
- Again, dropout was selected and 20% of the data was forgotten.
- Batch normalization was applied, and the data were reduced to 1—dimensional with the flattening process.
- Three different fully connected layers were employed. Their number of neurons was determined as 512, 256, and 128.
- In the last layer, the Sigmoid activation function was used, and the data were classified.
- Binary-cross entropy was used for the loss of the model and the model was optimized with the RMSProp optimization algorithm.
- The training process was carried out with 500 epochs.
- Seventy-five percent of the data was used for training, 15% for validation, and 15% for testing.

The results of the classification process are given in Table 4.

**Figure 3:**
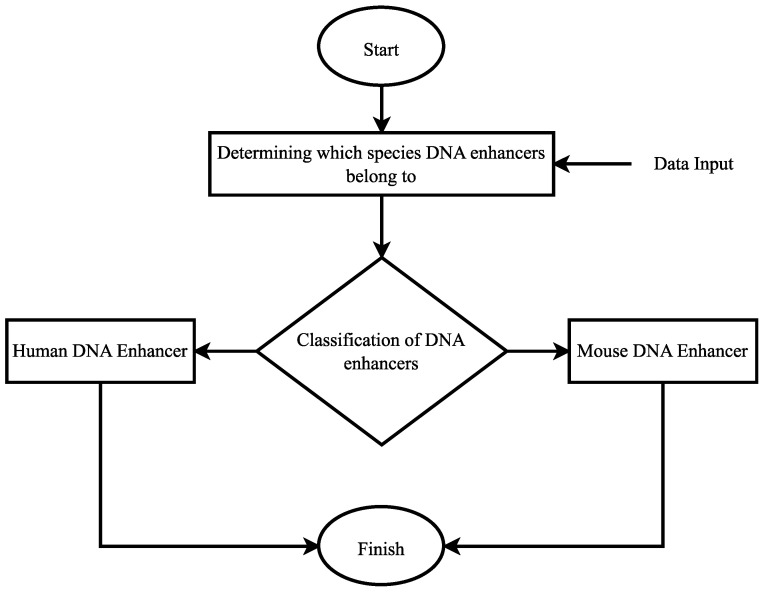
Flow chart of the algorithm for the first scenario.

When the results in Table 4 were examined, only the accuracy score of the proposed method was over 90% and became 92.16%. In addition, the highest precision, recall, and F1-score were obtained with the proposed method, and these values were 89.76%, 94.11%, and 91.88%, respectively. The AUC score of the proposed BFDNA method was obtained as 0.85. The closest accuracy score to the proposed method was obtained by EIIP and atomic number DNA encoding schemes and the results were obtained as 89.14% and 86.61%, respectively. The precision score of the EIIP method was 87.07%, the recall value was 90.61%, and the F1-score value was 88.80%. In addition, the highest AUC score was obtained with the EIIP, and the result was 0.87. The AUC score of the atomic number DNA encoding scheme was obtained as 0.84. In addition, the precision, recall, and F1-score values of this method were found to be 85.36%, 87.28%, and 86.31%, respectively. The most ineffective classification process was obtained by an integer number DNA encoding scheme. With this method, all evaluation criteria except the AUC score were below 80%. The AUC score was calculated as 0.82 with this method. When a comparison was made according to the CSI values, a value of 0.5429 was obtained with the integer number method, whereas the values of 0.7264 and 0.7768 were calculated with the atomic number and EIIP methods, respectively. The highest CSI value was obtained with the proposed BFDNA, and the result was 0.8387. A CSI value of 1 indicates a great classification. The fact that the proposed method also produced a score close to 1 showed that this method performed an almost perfect classification. In addition, the MCC value of only the integer number DNA encoding method remained below 0.70 and the result was 0.5397. On the other hand, atomic number and EIIP DNA encoding methods showed MCC scores above 0.70. The highest MCC score was obtained with the recommended BFDNA method, and the result was 0.8440. The closer the MCC score is to 1, the more effective the classification. Considering the MCC results, it has been observed that DNA encoding methods other than integer number DNA encoding were effective. When observations were made according to the Kappa results, it was observed that the integer number DNA encoding method was the most unsuccessful. With this method, a Kappa coefficient of 0.5393 was obtained and moderate agreement was observed between the classes. In addition, Kappa coefficient values of atomic number and EIIP DNA encoding methods remained between 0.61 and 0.80. Therefore, it has been shown that there is a substantial agreement between the classes for these two methods. On the other hand, the most effective Kappa coefficient was obtained with the proposed BFDNA DNA encoding method and the result was 0.8431. This showed that there was almost a perfect agreement between the classes. The results in Table 4 are the results from the first scenario. The flow chart of the algorithm for the first scenario is shown in Figure 3.

**Figure 4:**
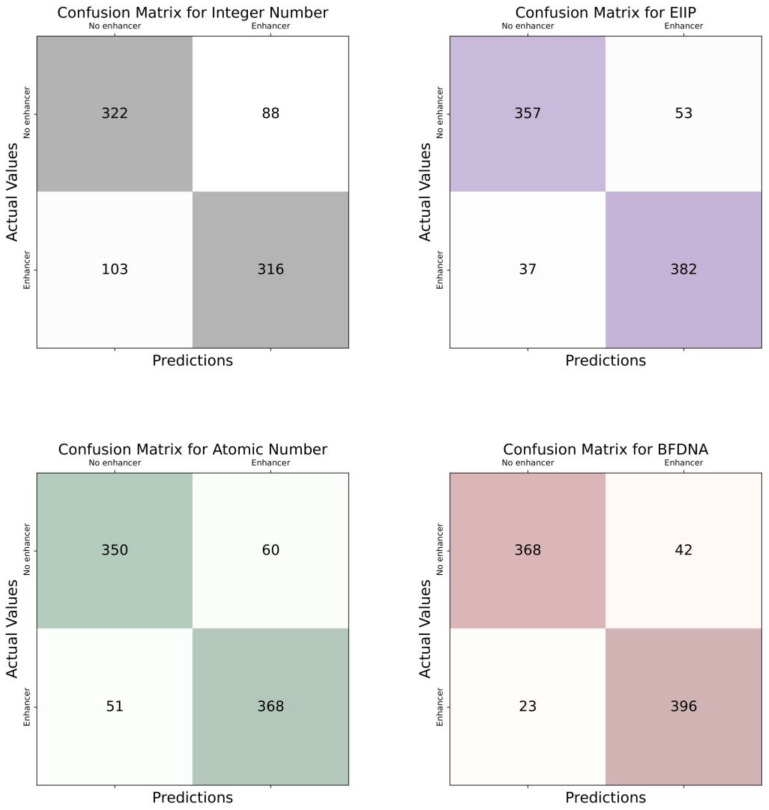
Confusion matrices of DNA encoding schemes for the first scenario.

**Figure 5:**
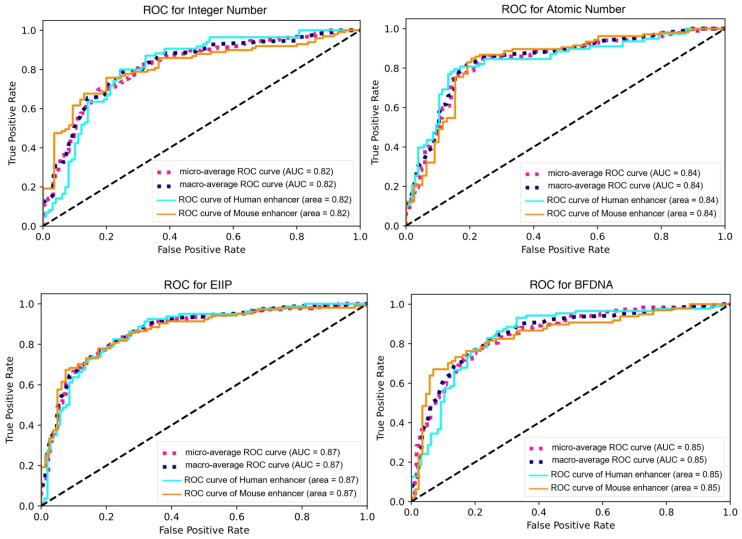
ROC curves of DNA encoding schemes for the first scenario.

According to the scenario given in Figure 3, data is fed into the system in the first step and arranged according to their labels. There are two different labels in this scenario. These are “Human enhancer” and “Mouse enhancer” labels. Then, these labels were trained by applying the deep learning model. At the end of the training process, a blinded data set was used, and the model was tested. In the testing phase of the model, classification was carried out according to the determined labels. According to the labels, the distinction was made whether the sequences belonged to humans or mice, and the scenario was finished. Since there are two different labels, binary classification was performed. The main purpose of this scenario is to classify the DNA enhancers of these two species. Moreover, the confusion matrix and ROC curves of each DNA encoding method are given in Figure 4 and Figure 5, respectively, to show their performance.

The pink and dark blue dots in Figure 5 represents micro-average ROC and macro-average ROC values, respectively. Micro-average ROC is the sum of the true positive rate divided by the sum of the false positive rate. In other words, each class will have a weightage. On the other hand, macro-average requires computing the metric independently for each class and then taking the average over them, thus treating all classes equally a priori.

### 4.2. Predicting DNA Enhancers

In this scenario, it was estimated whether there was a DNA enhancer and if so, which species it belonged to was predicted. Both the proposed DNA encoding scheme and other DNA encoding schemes have been applied in this scenario as well. BiLSTM deep learning model was used in this classification process and the performances of DNA encoding schemes were determined by accuracy, precision, recall, F1-score, CSI, G-mean, MCC, Kappa coefficient, and AUC scores. The hyperparameters of the developed BiLSTM method are as follows:

- DNA sequences used for training data in the input layer were evaluated.
- In the second layer, 128-unit BiLSTM was used, and the activation function was determined as SeLU.
- Dropout was done and 15% of the data was discarded.
- Then the 64-unit BiLSTM was used and the activation function SeLU was chosen.
- Again, dropout was selected and 20% of the data was forgotten.
- Batch normalization was applied, and the data were reduced to 1—dimensional with the flattening process.
- Two different fully connected layers were employed. Their number of neurons was determined as 256 and 128.
- In the last layer, the Softmax activation function was used, and the data were classified.
- Categorical-cross entropy was used for the loss of the model and the model was optimized with the Adam optimization algorithm.
- The training process was carried out with 500 epochs.
- Seventy-five percent of the data was used for training, 15% for validation, and 15% for testing.

The results of the classification process are given in Table 5.

**Figure 6:**
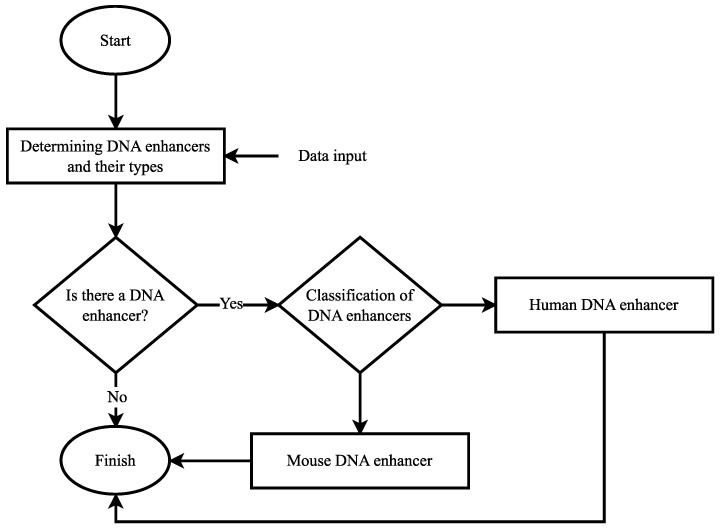
Flow chart of the algorithm for the second scenario.

When the results in Table 5 were examined, only the accuracy score of the proposed method was over 80% and became 84.59%. In addition, the highest precision, recall, and F1-score were obtained with the proposed method and these values were 85.64%, 80.35%, and 82.91%, respectively. The AUC score of the proposed BFDNA method was obtained as 0.92. The closest accuracy score to the proposed method was obtained by the EIIP DNA encoding scheme and a value of 77.80% was observed. The precision score of the EIIP method was 79.10%, the recall value was 73.11%, and the F1-score value was 76.00%. In addition, the AUC score was 0.90. The accuracy score of the integer number DNA encoding scheme was 73.68% and it was observed as the third effective method. The precision score of this method was 74.58%, the recall score was 68.96%, and the F1-score value was 71.66%. The AUC score was observed as 0.89. The most ineffective classification process was obtained by atomic number DNA encoding scheme. With this method, all evaluation criteria except the AUC score were below 80%. The AUC score was calculated as 0.81 with this method. In addition to these results, when the CSI values were examined, it was observed that the DNA encoding methods could not perform a very effective prediction. The CSI values of the integer number and atomic number DNA encoding methods remained below 0.5 and the results were 0.4354 and 0.3281, respectively. Although the EIIP method showed a CSI score above 0.5, it could not perform a very effective prediction process. However, the highest CSI score was obtained with the proposed BFDNA DNA encoding method and the result was 0.6599. As the CSI value approached 1, the prediction processes of the model were great. None of the four DNA encoding methods came close to 1. However, the most effective CSI score was obtained with the proposed method. Very high scores were not observed in MCC values either. The MCC value of the atomic number DNA encoding method was 0.4968, whereas this value was 0.5865 in the integer number DNA encoding method. The MCC value of the EIIP DNA encoding method was 0.6462. In contrast to these DNA encoding methods, only the proposed BFDNA DNA encoding method exceeded 0.7, resulting in 0.7467. It is a known fact that the model becomes effective as the MCC value approaches 1. In this sense, when the results were examined, it was observed that the most effective method was the proposed method. In addition, when DNA encoding methods were analyzed according to Kappa coefficients, it has been observed that there was moderate agreement between classes in atomic number and integer number DNA encoding methods. On the other hand, the existence of a substantial agreement with the classes was observed in EIIP and proposed BFDNA DNA encoding methods. The results in Table 5 are the results from the second scenario. The flow chart of the algorithm for the first scenario is shown in Figure 6.

**Figure 7:**
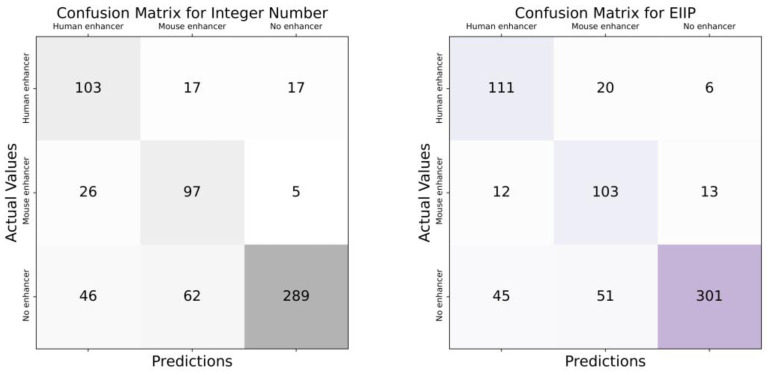

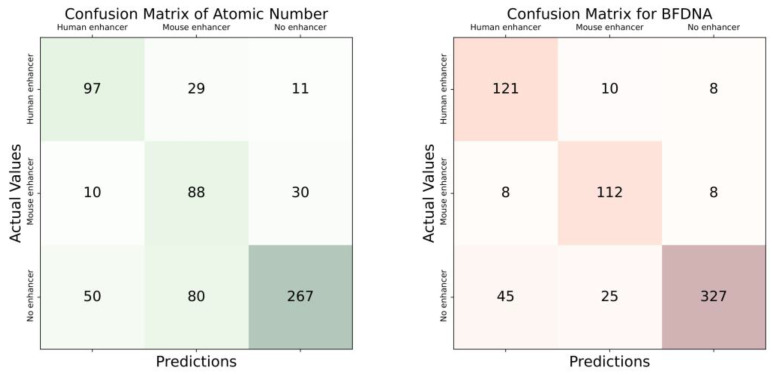
Confusion matrices of DNA encoding schemes for the second scenario.

**Figure 8:**
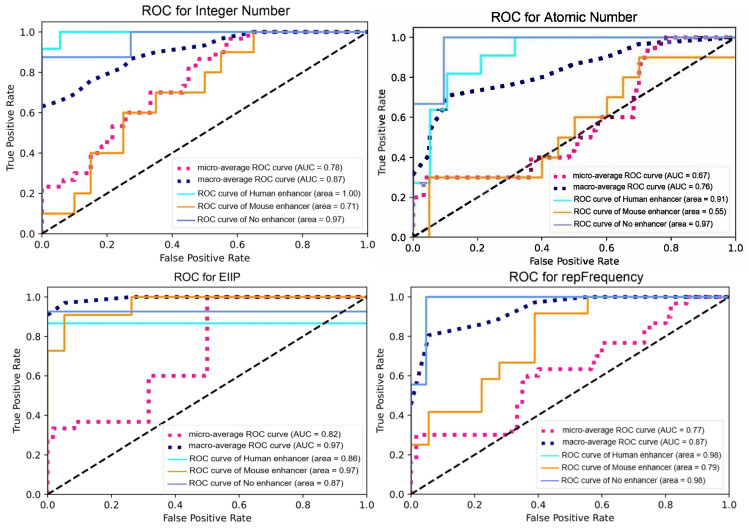
ROC curves of DNA encoding schemes for the second scenario.

In the scenario given in Figure 6, DNA sequences are first given to the system. There are three different class labels in this scenario. These are the “Human DNA enhancer”, “Mouse DNA enhancer”, and “No enhancer”. Then, these data were trained with a deep learning model. The performance of the model was then tested on a blinded dataset. At the test stage, it was determined whether there was a DNA enhancer in the DNA sequences. If there is no enhancer, the classification label for this part was predicted as “No enhancer”. If there is a DNA enhancer, a distinction has been made as to which type this enhancer belongs to. When the classification process of all sequences in the blind dataset was completed, the scenario was terminated. Since there are three different class labels multi-class classification was performed in this scenario. Furthermore, the confusion matrix and ROC curves of each DNA encoding method are given in Figure 7 and Figure 8, respectively, to show their performance.

### 4.3. Discussion

When the findings in Table 4 (first scenario) are examined, it is seen that the most effective prediction process is performed with the proposed BFDNA method. Other DNA encoding schemes, although exhibiting accuracy scores close to BFDNA, failed to pass. Although the highest accuracy score in the classification process according to the first scenario was obtained with the BFDNA method, successful results were also demonstrated with other methods. When compared according to the AUC scores, even if the most successful classification was made with EIIP, all methods produced a result above 0.80, and they performed great classification. In the findings in Table 5 (second scenario), the results decreased slightly. However, the most successful classification process was obtained with the proposed BFDNA encoding scheme. The closeness of accuracy seen in the first scenario was not observed in this scenario. However, when observing by AUC score, integer number and atomic number methods performed great classification, whereas EIIP and proposed BFDNA methods performed outstanding classification.

One of the biggest problems of the atomic number method is that it produces different results in various coding schemes [45]. Although it is a fixed method, the fact that coding schemes give different results leads to uniformity in research. These disadvantages may have caused the atomic number to be ineffective. The integer number DNA encoding scheme is one of the most used methods in studies with artificial neural networks. The biggest reason for this is that the mean is zero and the deviations are symmetrical [16]. Such symmetric and complementary features are effective for training data and extracting features. These achievements may have caused the integer number DNA encoding scheme to become more effective as the amount of data increased. In the EIIP method, real numbers are used, unlike integers and atomic numbers. This is an advantage that makes scientific calculation easy. Because of this advantage, it is effectively used in GSP studies that use neural networks and waveform transforms to show the pseudo-potential properties of nucleotide sequences [46,47]. In this study, the EIIP method was effective for both scenarios and performed more successful classification than both integer number and atomic number methods. The most successful classification process was obtained with the proposed BFDNA encoding scheme in two scenarios. One of the biggest reasons for this is that the proposed method has a dynamic structure. Other DNA encoding schemes have a static nature. The length of the sequence or the location of the bases does not affect the encoding process. However, this is not the case in the proposed BFDNA method. The proposed method varies according to the sequence and exhibits an adaptive presentation. In addition, the proposed BFDNA method, like the EIIP method, consists of real numbers. This is a factor that facilitates scientific computing, as in EIIP. The fact that these two methods are the most effective for both scenarios supports this.

In addition, the results obtained in this study were compared with other studies in the literature and the performance of the proposed method was demonstrated. In Table 6, these comparison results are given and interpreted.

When the results in Table 6 are examined, it is seen that the study performed with the proposed method has an accuracy of over 90%. The developed method was observed as the third most effective method among the compared studies. When studies [13] and [14] were examined, the accuracy scores were higher than the proposed method. The deep learning models used in the mentioned studies differ from the deep learning model used in this study. This may be the reason why the accuracy score was high in those studies. In addition, the word embedding method was used in the study [13]. The word embedding method is also a method frequently used in NLP studies. Since the developed model was inspired by the approach used in NLP studies, a performance close to the one in [13] was achieved. In the study [14], chemical properties were used. In the study, nine different chemical features were obtained for each DNA sequence. This resulted in the formation of a large number of sample data for a DNA sequence. The number of data is of great importance in deep learning studies. The large number of data had a positive effect on the deep learning model. The proposed method was more effective than the results of studies [10,11]. One of the reasons for this may be the DNA encoding methods used in those studies. In addition, selected deep learning algorithms directly affect this performance. However, it should be noted that the datasets used in the mentioned studies and the dataset used in this study are different. In bioinformatics studies, there is no standard gold data set, as in most artificial intelligence studies. This causes the comparison to be difficult most of the time and conducive to the inability to interpret the results properly. However, it is a fact that this proposed method showed as effective results as the existing methods in the literature. This shows that the method can be used effectively in other DNA studies.

Despite these achievements, there are several disadvantages in this study. These disadvantages can be identified as:

- Studies with genomic sequences vary greatly according to the numerical methods used. Although the deep learning method used in this study was the same, the results were different from each other. The lack of a standard method and the fact that the results vary according to the encoding methods cause the studies in this field to be limited and to be interpreted as unhealthy.
- Furthermore, mouse and human DNA enhancers are currently scarce. The increase in this number over time may affect the results obtained in this study positively or negatively, and accordingly may cause the results to change. A new analysis with an increase in the number of data will be more effective in evaluating the performance of both the proposed method and other methods.
- It is important to use the proposed method in other DNA analysis studies and to interpret the results to be obtained there. In this way, the performance of the proposed method can be demonstrated in detail.
- In addition, only BiLSTM deep learning model was used in this study. There are many deep learning methods. These DNA encoding schemes need to be analyzed with other deep learning algorithms and the results should be interpreted. In this way, more effective results can be obtained.
- No feature extraction was performed in the study. The use of different signal processing methods (DWT (discrete wavelet transform), FFT, EMD (empirical mode decomposition), VMD (variational mode decomposition), etc.) can be instrumental in obtaining more effective features and observing more successful results.
- Furthermore, optimization algorithms were not used in the study. Performing the optimization process can improve results and increase the performance of DNA encoding schemes.
- As seen in the ROC curve in Figure 8, the data set contains insufficient data. In this case, it may cause two different problems in the model: overfitting or underfitting. Obtaining, examining, and interpreting DNA sequences takes time and causes a difficult process. Therefore, the emergence of insufficient data is a common problem in bioinformatics studies [48,49]. There is such a problem in this study. Researchers need to consider this situation.
- In addition to the overfitting problem, approaches such as ensemble learning, transfer learning, and data duplication (synthetic data) are generally used for insufficient data. The insufficient data problem observed in this study can be addressed by using one or more of these approaches. Although each of these approaches has advantages and disadvantages, these approaches also need to be evaluated for further studies.
- In such cases, overfitting is generally observed. In the case of overfitting, the model memorizes patterns in the data. In order to avoid the overfitting problem, options such as reducing the network capacity, using regularization methods (L1 and L2), and placing dropout layers are generally used. For the second scenario in this study, although the network capacity was reduced and the dropout layer was used, this problem could not be avoided. The use of regularization methods or other approaches may prevent this problem.
- In order to prevent the overfitting problem, early stopping and pruning approaches can be used in addition. However, these approaches also have several disadvantages. Early stopping puts the artificial intelligence model’s training phase on hold before it can learn about the data noise. Nevertheless, if the timing is not set properly, the model will still not produce reliable results. The process of feature selection, also known as pruning, identifies the most crucial features in the training set and gets rid of the rest. Identifying effective features in this approach takes time and is often a tedious process.
- In addition, this problem can be avoided by using cross-validation. Although the number of labels (classes) of the data set used in the study is low, there are approximately 1000 features for each label. In short, each DNA sequence contains at least 1000 bases. The implementation of the cross-validation process takes time and increases the processing load. This approach can also be preferred on more powerful hardware and the performance of the developed DNA encoding method can be interpreted in a healthier way.
- In addition, the implications obtained from this study can be summarized as follows:
- Other cis-regulatory elements including promoters, insulators, and silencers can be predicted using the suggested BFDNA encoding technique. Experimental approaches are often preferred to determine cis-regulatory elements [50]. However, using experimental approaches takes time and is costly [51]. With this study and similar studies, it has been shown that cis-regulatory elements can be determined by computational approaches rather than experimental approaches.
- The proposed approach can also be used generically for research involving whole genomic sequencing, making it useful for both academics and healthcare professionals. In order for genomic sequences to be analyzed by computational methods, sequences must be converted to numerical expressions. There are various DNA encoding methods in the literature. In this study, some of these methods are included and their performances in these methods are evaluated. With this study, a novel DNA encoding method has been proposed in the literature. This method can be used not only to predict DNA enhancers, but also for genomic sequencing research (prediction of intron-exon regions, STR analysis, phylogenetic analysis, species identification, etc.).
- One of the biggest achievements of this study is that the developed BFDNA DNA encoding method has a dynamic structure compared to other methods. In all other approaches, the methods use a dynamic structure. Even if the length of the DNA sequence is different or the locations of the bases are different, the bases in the DNA sequence always take the same value. To give a simple example, in the atomic number DNA encoding method, the A base takes the value 70, regardless of the length of the DNA sequence or the location of the base. However, this is not the case in the proposed BFDNA method. Since this approach uses the length of the DNA sequence and the repetition frequency of the bases, there is no fixed value. This has resulted in the approach being adaptive and different from other DNA encoding methods.
- In studies on most DNA sequences, it has been observed that the chemical properties of DNA sequences are also used [52,53]. However, various experimental applications are used to determine these chemical properties. As this requires experimental equipment, it is both costly and time-consuming. With this study, it has been shown that DNA encoding methods used in computational approaches can also be effective in studies on DNA sequencing. When DNA enhancer studies were examined, it was observed that some studies focused on the chemical properties of DNA sequences [54,55,56,57,58]. When the performances in those studies were examined, it was observed that the accuracy scores ranged between 41.7% and 78%. In this study, only the integer number DNA encoding method was within this range, and an accuracy score of 77% was obtained. All remaining DNA encoding methods showed accuracy scores of over 85%. Moreover, the proposed BFDNA DNA encoding method achieved a high accuracy score of 92.16%. These results showed that computational features can also be effective.

## 5. Conclusions

In this study, a novel DNA encoding method called BFDNA has been proposed and applied to analyze the DNA enhancers of both humans and mice using data taken from the Vista enhancer browser data set with BiLSTM deep learning model. It has been observed that the proposed BFDNA encoding method is effective in predicting DNA enhancers. Moreover, the results of the proposed BFDNA encoding method were compared with other DNA encoding methods in the literature. A deep-learning-based DNA enhancer framework consisting of BiLSTM and BFDNA proved to be more effective in terms of accuracy, precision, recall, F1-score, AUC score, kappa, and MCC compared to the other DNA encoding methods. On account of this, this study introduces an intelligent computational model to identify and predict the DNA enhancers and their strength. Additionally, the proposed BFDNA encoding method can be employed to predict other cis-regulatory elements including insulators, promoters, and silencers. The results obtained from this study showed that the proposed BFDNA method can be used in various other DNA studies. In future studies, researchers will be able to use this developed method in STR analysis studies, phylogenetic analysis, prediction of intron and exon regions, and various DNA sequencing studies. Finally, this study demonstrates the performance of computational approaches and shows that these approaches can be more effective than experimental approaches. This may be an alternative to experimental approaches in future studies and may be more efficient in terms of cost, time, and laboratory equipment.

## Figures and Tables

**Table 1:** Comparison of DNA encoding schemes.

First DNA Sequence	Integer Number	Atomic Number	EIIP	BFDNA
[CATCG]	[3 1 4 3 2]	[58 70 66 58 78]	[0.134 0.126 0.133 0.134 0.081]	[0.4 0.2 0.2 0.4 0.2]
**Second DNA Sequence**				
[CGAAT]	[3 2 1 1 4]	[58 78 70 70 66]	[0.134 0.081 0.126 0.126 0.133]	[0.2 0.2 0.4 0.4 0.2]

**Table 2:** Interpretation of AUC scores [40].

AUC Score	Explanation
0.00–0.49	No distinction
0.50–0.69	Poor classification
0.70–0.79	Acceptable classification
0.80–0.89	Great classification
0.90–1.00	Outstanding classification

**Table 3:** Interpretation of Kappa coefficients [44].

Kappa Coefficient	Explanation
0.00	No agreement
0.10–0.20	Slight agreement
0.21–0.40	Fair agreement
0.41–0.60	Moderate agreement
0.61–0.80	Substantial agreement
0.81–0.99	Almost perfect agreement
1.00	Perfect agreement

**Table 4:** Performance of DNA encoding schemes in predicting human and mouse DNA enhancers.

DNA Encoding Scheme	Accuracy	Precision	Recall	F1-Score	CSI	G-Mean	MCC	Kappa	AUC Score
Integer number	76.96%	78.53%	75.76%	77.12%	0.5429	0.7698	0.5397	0.5393	0.82
Atomic number	86.61%	85.36%	87.28%	86.31%	0.7264	0.8663	0.7323	0.7321	0.84
EIIP	89.14%	87.07%	90.61%	88.80%	0.7768	0.8920	0.78.33	0.7826	0.87
**BFDNA**	**92.16%**	**89.76%**	**94.11%**	**91.88%**	**0.8387**	**0.9224**	**0.8440**	**0.8431**	**0.85**

**Table 5:** Performance of DNA encoding schemes in predicting DNA enhancers and their species.

DNA Encoding Scheme	Accuracy	Precision	Recall	F1-Score	CSI	G-Mean	MCC	Kappa	AUC Score
Integer number	73.68%	74.58%	68.96%	71.66%	0.4354	0.5416	0.5865	0.5731	0.89
Atomic number	68.27%	68.93%	64.38%	66.58%	0.3281	0.4597	0.4968	0.4836	0.81
EIIP	77.80%	79.10%	73.11%	76.00%	0.5221	0.6060	0.6462	0.6340	0.90
**BFDNA**	**84.59%**	**85.64%**	**80.35%**	**82.91%**	**0.6599**	**0.7104**	**0.7467**	**0.7394**	**0.92**

**Table 6:** Comparison of this study and some studies in the literature.

Reference	DNA Encoding Method	Classification Model	Accuracy
[10]	k-mer	DBN	92.00%
[11]	Binary hot encoding	Hybrid	89.70%
[13]	Word embedding	Hybrid	93.95%
[14]	Chemical features	DNN	96.82%
**This study**	**BFDNA**	**BiLSTM**	**92.16%**

## Data Availability

Publicly available dataset was analyzed in this study. This data can be found here: https://enhancer.lbl.gov/.
